# User-testing guidelines to improve the safety of intravenous medicines administration: a randomised in situ simulation study

**DOI:** 10.1136/bmjqs-2020-010884

**Published:** 2020-06-30

**Authors:** Matthew D Jones, Anita McGrogan, D K Raynor, Margaret C Watson, Bryony Dean Franklin

**Affiliations:** 1 Department of Pharmacy and Pharmacology, University of Bath, Bath, UK; 2 School of Healthcare, University of Leeds, Leeds, UK; 3 Luto Research, Leeds, UK; 4 Strathclyde Institute of Pharmacy and Biomedical Sciences, University of Strathclyde, Glasgow, UK; 5 Centre for Medication Safety and Service Quality, Imperial College Healthcare NHS Trust, London, UK; 6 Department of Practice and Policy, UCL School of Pharmacy, London, UK

**Keywords:** clinical practice guidelines, medication safety, nurses, patient safety

## Abstract

**Background:**

User-testing and subsequent modification of clinical guidelines increases health professionals’ information retrieval and comprehension. No study has investigated whether this results in safer care.

**Objective:**

To compare the frequency of medication errors when administering an intravenous medicine using the current National Health Service Injectable Medicines Guide (IMG) versus an IMG version revised with user-testing.

**Method:**

Single-blind, randomised parallel group in situ simulation. Participants were on-duty nurses/midwives who regularly prepared intravenous medicines. Using a training manikin in their clinical area, participants administered a voriconazole infusion, a high-risk medicine requiring several steps to prepare. They were randomised to use current IMG guidelines or IMG guidelines revised with user-testing. Direct observation was used to time the simulation and identify errors. Participant confidence was measured using a validated instrument. The primary outcome was the percentage of simulations with at least one moderate-severe IMG-related error, with error severity classified by an expert panel.

**Results:**

In total, 133 participants were randomised to current guidelines and 140 to user-tested guidelines. Fewer moderate-severe IMG-related errors occurred with the user-tested guidelines (n=68, 49%) compared with current guidelines (n=79, 59%), but this difference was not statistically significant (risk ratio: 0.82; 95% CI 0.66 to 1.02). Significantly more simulations were completed without any IMG-related errors with the user-tested guidelines (n=67, 48%) compared with current guidelines (n=26, 20%) (risk ratio: 2.46; 95% CI 1.68 to 3.60). Median simulation completion time was 1.6 min (95% CI 0.2 to 3.0) less with the user-tested guidelines. Participants who used user-tested guidelines reported greater confidence.

**Conclusion:**

User-testing injectable medicines guidelines reduces the number of errors and the time taken to prepare and administer intravenous medicines, while increasing staff confidence.

**Trial registration number:**

researchregistry5275.

## Introduction

Medication errors are a major cause of avoidable patient harm worldwide, with an estimated annual cost of $42 billion.^1^ An estimated 237 million medication errors occur annually in England, 28% with potential to cause harm.^2^ Intravenous medicines are complex to prepare and more prone to error,^3 4^ with 35%–48% of intravenous doses erroneous in some way.^3 4^

Patient safety incidents have numerous causes, one of which is written guidance for health professionals that is contradictory, incomprehensible or of poor quality.^5 6^ Specifically, medication errors have been caused by difficulty finding relevant, unambiguous information in guidelines.^7–10^ Two studies recommended that user-testing might improve medicines guidelines for health professionals and thus improve safety,^11 12^ where user-testing assesses whether potential users of a document can find and understand important information. Problems are identified and potential solutions tested iteratively until the document is shown to perform well.^11 13 14^

User-testing has been shown to increase the amount of information found and understood by doctors using Summaries of Product Characteristics (SPC),^11^ and nurses using injectable medicines guidelines.^12^ Studies of patient-facing medicines information, evidence summaries and infection control guidelines have also shown better understanding and faster reading time following user-testing.^13 15–17^ However, no published study has investigated whether health professionals using user-tested guidelines make fewer medication errors, and thus provide safer patient care.

Our aim was to investigate the effectiveness of user-testing of medicines guidelines for reducing medication errors. We selected the administration of intravenous medicines by hospital nurses using the UK’s National Health Service (NHS) Injectable Medicines Guide (IMG),^18^ due to the increased risk of medication errors with this route of administration.^3 4^ The IMG is designed to give guidance on the correct procedures for the preparation and administration of over 350 intravenous medicines and is accessed online approximately 3 million times per year.^18^ The majority of these users are nurses preparing and administering intravenous medicines in clinical areas, who typically refer to the IMG as they work through each step of this process.^12^ However, a recent user-testing study identified that in 36 of 340 cases, nurses were unable to find or understand important information in the current IMG.^12^ After user-testing, numerous revisions were made to the guidelines, including provision of equations and tables to support dose, dilution and infusion rate calculations, additional subsections, and clearer wording and formatting (online supplementary file 3). Following these changes, the number of instances of nurses being unable to find or understand important information decreased to 3 of 340 cases. Therefore, the specific objective of the present study was to compare the frequency of medication errors made by nurses using current and user-tested IMG guidelines during the preparation and administration of an intravenous medicine.

10.1136/bmjqs-2020-010884.supp3 Supplementary data

## Methods

### Study design

We conducted a single-blind, randomised parallel group in situ simulation experiment with a 1:1 allocation ratio. Participants prepared and administered a simulated intravenous infusion of voriconazole, with observation used to identify medication errors. In situ simulation involves a simulated episode of patient care integrated into the clinical environment with participants who are on-duty health professionals. It is useful when it is not feasible to test interventions during routine care, but participants still experience the pressures and distractions present in the clinical environment.^19^

### Participants and recruitment

Participants were on-duty nurses or midwives registered with the Nursing and Midwifery Council, who were authorised to prepare and administer intravenous medicines in their hospital and had done so during at least 50% of working shifts during the previous 6 months (or since authorisation if more recent) (self-assessed). They were recruited from four NHS acute hospital trusts between January and July 2019. The managers of a range of different clinical departments (including medical and surgical inpatient wards, intensive therapy units, emergency departments, postanaesthetic care units and birthing units) agreed suitable times for simulations to take place and circulated written information about the study to their nursing staff. This informed potential participants that the study was investigating how changes to guidelines affect patient safety. At the agreed times, the researcher approached eligible staff currently working in the area and invited them to take part. Participation was voluntary and only occurred at a safe time for the participant’s patients. Therefore, to facilitate recruitment, the researcher waited in each department until all interested staff had taken part.

### Definitions

An error was defined as any deviation from the simulated medication order, the hospital’s policies or the IMG guidelines.^3 20 21^ Up to 30 errors were possible in a single simulation, regardless of the version of the guidelines used (structured observation forms, online supplementary file 2). An ‘IMG-related error’ was an error in a process that required use of information from the IMG. There were 11 types of IMG-related error, including use of incorrect fluids, fluid volumes or technique for reconstitution or dilution, administration of an incorrect dose or at an incorrect rate and not flushing the intravenous cannula in accordance with hospital policy. All other errors were ‘non-IMG-related errors’, with five error types including selection of the wrong medicine, use of expired ingredients and breach of the hospital aseptic technique policy. Online supplementary file 3 presents definitions of error types. Deviations from procedures designed to reduce the likelihood of a subsequent error were not themselves included as errors, for example, labelling and documentation errors.^21^

10.1136/bmjqs-2020-010884.supp1 Supplementary data

10.1136/bmjqs-2020-010884.supp2 Supplementary data

### Simulated task

The simulation was carried out in the area of each participant’s usual clinical department designated for the preparation of intravenous medicines. This was most often a small room where medicines were stored and prepared, but empty bed spaces were used in some areas. These areas were still available to other users while the simulation was taking place.

Each participant worked alone to prepare one simulated intravenous infusion of voriconazole in response to an inpatient medication order for a 360 mg (6 mg/kg) adult loading dose, following the procedures they would normally employ. Voriconazole was chosen as it is classed as a high-risk medicine and requires a variety of intravenous procedures, including reconstitution, dilution and infusion at a controlled rate.^18^ Participants selected a vial from a box that contained 23 labelled placebo voriconazole vials (Dummy-Ject Powder C, MockMeds, Houston, Texas), 1 identical placebo vial labelled with another drug name and 1 identical vial of placebo voriconazole that had expired (as may occur in practice). Images of the vials and labels are shown in online supplementary file 2. All other equipment was taken from routine stock in the participant’s clinical area. Participants then administered this one dose to an intravenous training manikin arm (Multi-Venous, Laerdal Medical, Orpington, UK).

Participants were randomised to use one of two versions of the IMG guidelines for voriconazole on a laptop computer. The control group used the version of the guidelines used in practice (‘current guidelines’). The other group used a version of the guidelines revised through three rounds of user-testing with hospital nurses (‘user-tested guidelines’), as described above.^12^ Both versions contained the same information, presented in different ways (online supplementary file 1). To prevent participants using prior knowledge, the drug name ‘voriconazole’ was changed to ‘bathicillin’ in all study documents and labels. Brand names were also changed. The descriptions of the strength of one vial, its displacement volume and the required volume of reconstituting fluid were adjusted in the IMG guidelines to match the available placebo vials. References to the compulsory use of an infusion pump were removed, as this equipment was not consistently available. Therefore, participants could correctly administer an infusion using either an infusion pump or a gravity infusion set (infusion rate calculated from drip rate).

Participants were given no directions on how to use the guideline, but were asked to follow their usual practice. However, as ‘bathicillin’ was an unknown medicine for all participants, the allocated guideline was their only potential source of information on how to correctly prepare and administer the dose.

### Data collection

Initially, participant characteristics were documented to confirm eligibility. Then participants prepared and administered their dose while being observed by one researcher (MDJ) who noted preparation or administration errors on a structured form (online supplementary file 2). Observation is considered the most robust method for quantifying medication administration errors.^3 20 22 23^ The time taken to complete the simulation was recorded, from when the participant opened their IMG guideline to when they informed the researcher they would leave the simulated patient’s bedside after starting the infusion. After finishing the simulation, each participant completed a modified version of the Provider Decision Process Assessment Instrument (mPDPAI) questionnaire to measure their perceived degree of knowledge and uncertainty during the simulation.^24^ Modifications included removing questions 6, 8, 9 and 10 from the original instrument, as these related to concepts not relevant in this context. The remaining questions were reworded to focus on a series of decisions on how to prepare and administer voriconazole (online supplementary file 2). To investigate the psychometric properties of the mPDPAI, participants also answered quality and satisfaction validation questions.^24^ Finally, each participant was shown both versions of the IMG guidelines and asked to review them for as long as they wished (typically 1–2 min) before stating which they preferred, using a 7-point scale ranging from strongly preferring the current guidelines to strongly preferring the user-tested guidelines (online supplementary file 2).

To investigate the reliability of the observations, 10% of simulations were recorded using a video camera (Akaso EK7000) mounted on the participant’s forehead. This recorded images of wherever the participant looked, including medicine preparation. Videos were viewed independently by a second researcher, using the same structured form to record errors.

### Error severity classification

The potential severity of each observed error was assessed using a validated method appropriate for situations where actual patient outcome is unknown.^3 4 25^ A panel was established, comprising two consultant physicians, three hospital pharmacists and two hospital nurses (minimum 10 years of experience). Each panel member was sent a brief description of each error and asked to score its potential clinical significance on a scale from 0 (no harm) to 10 (death). The panel was informed that incidents related to an adult weighing 60 kg prescribed a 6 mg/kg loading dose (360 mg) of voriconazole for administration by intravenous infusion over 2–3 hours. They were asked to assume that the patient was on a general medical or surgical ward with peripheral venous access. As a full clinical history was not available, they were asked to rate the potential clinical significance for a typical patient prescribed voriconazole. To investigate the validity of this process, the panel also scored 15 injectable medication errors with a known outcome from the literature (five each with known minor, moderate and severe outcomes).^26–30^ The mean panel score for each error was calculated and used to classify it as either a minor (mean score <3), moderate (mean score 3–7) or severe error (mean score >7).

### Outcomes and sample size

The primary outcome was the observed frequency of simulations with one or more IMG-related moderate-severe errors. Frequencies were expressed as percentages using the number of simulations considered to have one or more errors as the numerator and the total number of simulations as the denominator.^3 4^ Where multiple errors were observed within the same simulation, the severity of the most serious error was assigned to the simulation. Secondary outcomes were the frequency of simulations with one or more moderate-severe non-IMG-related errors, the frequency of simulations without any IMG-related errors and the frequency of simulations without any non-IMG-related errors, as well as the time taken to complete the simulation, a decisional conflict score (DCS) derived from mPDPAI responses (see the Analysis section) and guideline preference.

Assuming a frequency of moderate-severe IMG-related errors of 30% in the ‘current guidelines’ group,^3 4^ a clinically relevant reduction to 15% in the ‘user-tested guidelines’ group, a 5% significance level, 80% power and no clustering by hospital, a sample size of 121 participants/group was calculated.^31^ A power calculation accounting for clustering by hospital gave a sample size of 172 participants per group for four NHS trusts (based on similar assumptions and an intraclass correlation coefficient of 0.01). Therefore, a minimum sample size of 121 participants per group was adopted, with a maximum of 172 participants per group.

### Randomisation and blinding

The allocation sequence was generated by an independent researcher using an online blocked randomisation list generator (www.sealedenvelope.com/simple-randomiser/v1/lists) (block size of 10). Allocation was stratified by research site and participant experience (<5 or ≥5 years of accreditation for the administration of intravenous medicines). The researcher enrolled and then allocated participants immediately prior to their simulation using an online randomisation service (www.sealedenvelope.com). This generated a unique code, which identified the computer file containing the participant’s IMG guideline.

The researcher was blinded to participant allocation by the use of a privacy filter on the laptop computer screen. This permitted only a reader directly in front of the screen to see the information displayed. The laptop computer was angled away from the researcher, so he was unable to see the IMG guideline. Data analysis was conducted using blinded conditions.

### Analysis

Statistical analysis was performed using SPSS (V26) and STATA. Only data from completed simulations were included in the analysis. Participants were analysed in the group to which they were allocated. The inter-rater reliability between the live and video observations was quantified using Cohen’s kappa, with each step on the observation form constituting a data point (error vs no error).^32^ The inter-rater reliability of the severity scoring panel was calculated using Cronbach’s alpha.^33^ The validity of the scores was investigated by comparing the mean severity scores of the literature errors with their known outcomes.

The median time taken to prepare and set up the infusion in the two groups was calculated using the Kaplan-Meier estimator. This accounted for censored simulations, defined as participants being unable to finish the simulation without assistance from a colleague.

mPDPAI responses were used to calculate a modified DCS (mDCS) for each participant, following the methods described for the original PDPAI.^24^ Theoretically, mDCS could range from 8 to 40, with a higher score reflecting greater uncertainty while preparing and administering voriconazole. The psychometric properties of the mPDPAI were investigated using the methods used during the initial development of the PDPAI.^24^ Item homogeneity was evaluated by calculating Spearman’s rank correlation coefficients between each item and a revised mDCS calculated by removing that item from the total score. Interitem reliability was investigated by the calculation of Cronbach’s alpha. Construct validity was investigated by hypothesising that there would be negative correlations between the mDCS and participants’ self-rating of the quality of their dose of voriconazole, and between the mDCS and how participants would feel if every intravenous drug administration was like the simulation.^24^ This was investigated by calculating Spearman’s rank correlation coefficients between participants’ mDCS and their responses to the quality and satisfaction validation questions. It was also hypothesised that nurses who are more uncertain would take longer to complete the simulation. This was investigated by calculating the Spearman’s rank correlation coefficient between mDCS and time taken to complete the in situ simulation. Finally, it was hypothesised that nurses who are more uncertain were more likely to make an IMG-related error. This was investigated using a logistic regression of the presence or absence of IMG-related errors for each participant with the mDCS as a variable.

Responses to the guideline preference rating scale were compared between the two groups using a χ^2^ test. Due to small expected counts, responses for the categories ‘Strongly prefer current guidelines’, ‘Prefer current guidelines’ and ‘Somewhat prefer current guidelines’ were combined.

Multivariate analysis was carried out to determine the influence of the allocated IMG guideline on the study outcomes while adjusting for participant characteristics: first language (English or not), experience administering intravenous medicines (more or less than 5 years), current use of the IMG (regular or not) and NHS trust. The frequency of errors was investigated using logistic regression with model-based estimates used to convert the ORs to risk ratios, to aid interpretation. Simulation completion times were investigated using a Cox proportional hazards model and mDCS using multiple linear regression. For all outcomes, both mixed effects models (with NHS trust as the random effect and other characteristics as fixed effects) and fixed effects models were investigated. Models were checked for fit and results from the best fitting models presented.

## Results

**Figure 1:**
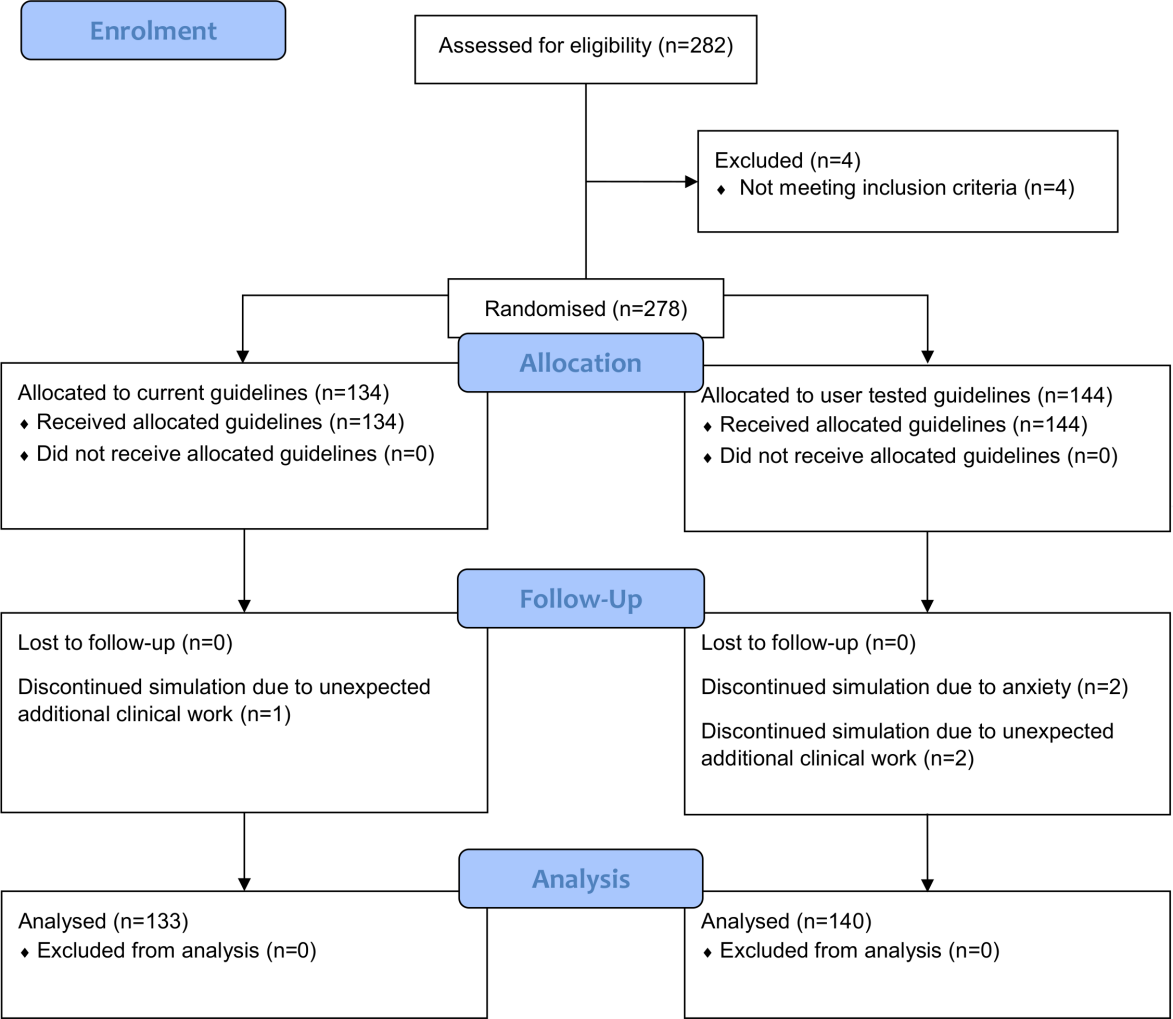
Flow diagram of participant progress through the phases of this randomised in situ simulation experiment.

In total, 273 participants completed the simulation before recruitment ended with all willing participants recruited and the minimum sample size achieved (figure 1). Two participants withdrew due to anxiety and three due to unexpected additional work. Participant characteristics were similar in the two groups (table 1). In total, 189 participants (69%) reported being regular users of the current guideline format. Some participants were observed to read their allocated guideline, gather equipment and plan their work before starting to prepare the dose, whereas other participants read the guideline concurrently with working through the task.

**Table 1:** Characteristics of participants who completed the simulation (n=273)

		Current guidelines (n=133)	User-tested guidelines (n=140)
Number of female participants (% within group)		116 (87)	125 (89)
Mean age (SD)		35.9 (10.3)	35.3 (10.7)
Median years nursing experience (IQR)		7.0 (4.0–14.0)	8.5 (3.0–14.8)
Median years authorised to give intravenous medicines (IQR)		6.5 (3.0–12.0)	6.8 (3.0–13.4)
Median percentage of shifts in which intravenous medicines administered (IQR)		100 (90–100)	100 (95–100)
Number with English as first language (% within group)		109 (82)	105 (75)
Previous experience of the NHS Injectable Medicines Guide (% within group)	Regular user	91 (68)	98 (70)
	Past user	12 (9)	14 (10)
	Seen	21 (16)	14 (10)
	Not seen	9 (7)	14 (10)
Number of participants from each NHS trust (% within group)	1	43 (32)	43 (31)
	2	25 (19)	25 (18)
	3	36 (27)	41 (29)
	4	29 (22	31 (22)

NHS, National Health Service.

Cohen’s kappa for the comparison of live and video observations (28 simulations) was 0.90, indicating high agreement.^32^ There was high agreement among the expert panel (Cronbach’s alpha 0.93) regarding the clinical significance of errors. For 13 of 15 injectable medication errors with a known outcome from the literature, the mean potential clinical significance score was equivalent to their known outcome. For one error with a known severe outcome (equivalent to a mean potential clinical significance score >7), the mean potential clinical significance score was 6.6. For another with a known minor outcome (equivalent to a mean potential clinical significance score <3), the mean potential clinical significance score was 5.7. The item homogeneity, interitem reliability and construct validity of the mPDPAI were all high (online supplementary file 3).

In total, 1504 errors were observed (table 2) and 267 of 273 simulations (98%) included at least one error of any type. For the primary outcome, although a smaller proportion of the simulations with the user-tested guidelines (49%) included at least one moderate-severe IMG-related error than with the current guidelines (59%), the risk ratio of 0.82 (95% CI 0.66 to 1.02) did not indicate a significant difference (table 3). However, the proportion of user-tested guideline simulations without any IMG-related errors (48%) was significantly greater than with the current guidelines (20%), with a risk ratio of 2.46 (95% CI 1.68 to 3.60). This indicates that when using the user-tested guidelines, a simulation was more than twice as likely to have no IMG-related errors than when using the current guidelines. Risk ratios indicate no significant differences between the groups for non-IMG-related errors (table 3).

**Table 2:** Errors observed during simulation study, categorised by potential severity and error type

Error code*	Error type	Current guidelines (control) (n=133 simulations)				User-tested guidelines (n=140 simulations)			
		Minor	Moderate	Severe	Total	Minor	Moderate	Severe	Total
	**Number of IMG-related errors**								
I1	Wrong reconstituting fluid	0	0	0	0	0	0	0	0
I2	Wrong reconstituting fluid volume	1	0	0	1	0	0	0	0
I3	Dose discrepancy	13	16	0	29	4	6	0	10
I4	Wrong diluent	0	0	0	0	0	1	0	1
I5	Wrong diluent volume	0	6	0	6	0	0	0	0
I6	Incorrect technique (IMG related)	1	55	0	56	6	58	0	64
I7	Wrong route	0	0	0	0	0	0	0	0
I8	Flush error	0	12	0	12	0	12	0	12
I9	Rate discrepancy	10	30	0	40	1	12	0	13
I10	Infusion expiry error	0	0	0	0	0	0	0	0
I11	Other IMG-related error†	23	4	5	32	1	2	1	4
	Total IMG-related errors	48	123	5	176	12	91	1	104
	**Number of non-IMG-related errors**								
N1	Wrong medication	0	5	0	5	0	6	0	6
N2	Incorrect technique (non-IMG related)	64	34	0	98	69	52	0	121
N3	Non-aseptic technique	264	194	0	458	289	220	0	509
N4	Expired ingredient	0	5	0	5	0	2	0	2
N5	Other non-IMG-related error	10	0	0	10	9	1	0	10
	Total non-IMG-related errors	338	238	0	576	367	281	0	648

*Error codes cross-reference to the observation recording forms shown in online supplementary file 2 and online supplementary file 3, table 4.

†Other IMG-related errors consisted of participants who were not confident to finish the simulation without assistance from a colleague and composite errors where a participant gave the dose as a short injection, so it was both undiluted and administered too quickly.

IMG, Injectable Medicines Guide.

**Table 3:** Primary and secondary outcomes by group

	Current guidelines (control) (n=133)	User-tested guidelines (n=140)	Multivariate analyses*
Number of simulations with one or more moderate-severe IMG-related errors (% within group)	79 (59)	68 (49)	RR: 0.82†‡ (95% CI 0.66 to 1.02)
Number of simulations with one or more moderate-severe non-IMG-related errors (% within group)	109 (82)	124 (89)	RR: 1.09†§ (95% CI 0.98 to 1.20)
Number of simulations without any IMG-related errors (% within group)	26 (20)	67 (48)	RR: 2.46‡ (95% CI 1.68 to 3.60)
Number of simulations without any non-IMG-related errors (% within group)	11 (8)	6 (4)	RR: 0.45§ (95% CI 0.19 to 1.30)
Median minutes taken to prepare and set up infusion (95% CI)	13.0 (12.2 to 13.8)	11.4 (10.5 to 12.2)	HR: 1.45‡ (95% CI 1.11 to 1.89)
Median modified decisional conflict score (IQR)¶	27 (18–32)	16 (13–18)	MRC: −9.83‡ (95% CI −11.36 to −8.32)

*Multivariate analyses compare outcomes for the user-tested guidelines relative to outcomes for the current guidelines. They were adjusted for participant characteristics: first language (English or not), experience administering intravenous medicines (more or less than 5 years), current use of the IMG (regular or not) and National Health Service (NHS) trust.

†Only NHS trust included as covariate. Other participant characteristics did not improve fit of the model.

‡NHS trust included in model as random effect.

§NHS trust included in model as fixed effect.

¶The modified decisional conflict score (mDCS) can range from 8 to 40, higher numbers represent greater uncertainty.

IMG, Injectable Medicines Guide; MRC, multiple regression coefficient; RR, risk ratio.

**Figure 2:**
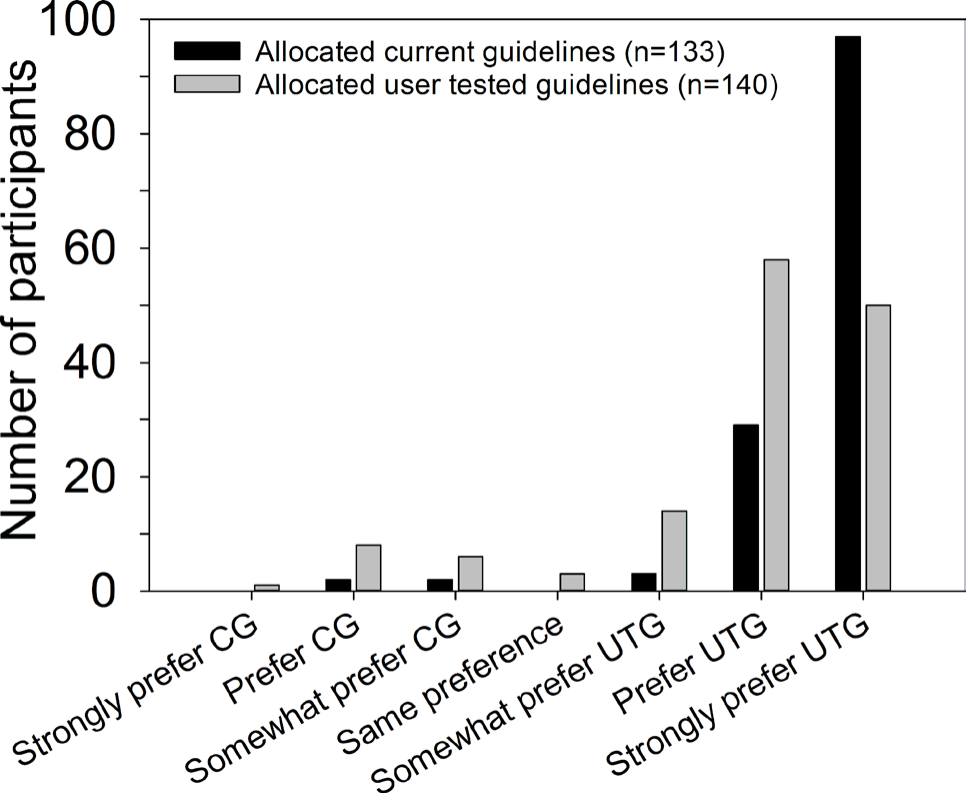
Number of participants expressing different levels of preference for either the current guidelines (CG) or user-tested guidelines (UTG).

Median completion time for the simulation was 1.6 min (95% CI 0.2 to 3.0) faster with the user-tested guidelines (table 3), indicating a statistically significant decrease in completion time. There was a statistically significant 45% increase in the chance of completing the simulation if using the user-tested guidelines compared with the current guidelines (HR 1.45, 95% CI 1.11 to 1.89). Online supplementary file 3, figure 3 shows the Kaplan-Meier curve for these data. The mDCS suggest that participants allocated the user-tested guidelines reported more confidence in their knowledge of how to prepare and administer intravenous voriconazole. The mDCS multiple regression coefficient indicates that on average, participants using the user-tested guidelines had an mDCS of 9.83 less than those using current guidelines, which is a substantial proportion of a scale that has a maximum range of 32 points (8–40). After comparing both versions of the guidelines, 129 participants allocated the current guidelines (97%) and 122 participants allocated the user-tested guidelines (87%) expressed at least some preference for the user-tested guidelines (figure 2). This difference between the two groups was statistically significant (p<0.001; χ^2^ test).

## Discussion

This study is the first to investigate whether user-testing of guidelines results in safer medication administration. There was no significant difference in the frequency of moderate-severe medication errors. However, use of user-tested guidelines more than doubled the probability of avoiding an IMG-related error during preparation and administration of an intravenous medicine on hospital wards. The procedure was also completed faster with user-tested guidelines, as participants were able to locate the information they required more quickly. Nurses felt more confident about their decisions when using user-tested guidelines and preferred them to the original version.

The guideline revisions likely to have contributed to the reduced frequency of errors after user-testing can be identified by considering the error types with the largest reduction (table 2). These include dose and rate discrepancies (including administration by short injection rather than infusion), suggesting that the improved support for dose, rate and dilution calculations in the user-tested guidelines (eg, equations and tables)^12^ at least partially contributed to safer and faster preparation and administration of the medicine. This is consistent with the findings of our previous study, where dose and rate calculation problems occurred more often with the current guidelines than with the user-tested guidelines.^12^ A common calculation error with the current guidelines was not accounting for displacement volume when measuring the volume of drug solution containing the prescribed dose. The user-tested guidelines helped prevent this error by providing an equation for this calculation. A common rate discrepancy with the current guidelines was to administer the infusion over 1 hour. The instructions stated ‘give over 1–3 hours (maximum rate 3 mg/kg/hour)’, and some participants used the first time listed (1 hour) rather than calculating the maximum infusion rate based on the second part of this instruction (3 mg/kg/hour=2 hours). The user-tested guidelines did not present this range of infusion times (which was only applicable to some doses) and instead provided a table of infusion lengths and an equation to support the calculation, thus preventing this error. This suggests that guideline authors should provide equations and tables to support health professionals making calculations. However, as different ways of presenting information may be successful in different contexts, the safety improvements suggested by this study will be best achieved through user-testing.

These results build on previous findings by demonstrating for the first time that user-tested guidelines can change the actions of nurses and thus result in fewer errors. In contrast, previous research has focused on health professionals’ comprehension of information. A randomised study found that a user-tested version of a Cochrane review improved participants’ satisfaction and correct comprehension of key results, while decreasing reading time.^15^ Similar results were obtained from a non-randomised study of user-tested infection control guidelines.^16^ The only previous studies of medicines guidelines have shown that user-testing improves the retrieval and comprehension of information from documents such as SPCs^11^ and the IMG used in this research.^12^

Among the strengths of this study are its blinded, randomised design and the ‘anonymisation’ of the drug name in the IMG guidelines to prevent use of prior knowledge. Data were collected from nurses working in their usual clinical environment, thus ensuring a high-fidelity simulation in which participants in both groups were exposed to typical work pressures and distractions. The validity and reliability of the error observation process, the potential clinical significance scores and the mPDPAI were also confirmed.

Limitations include use of simulation rather than the investigation of actual patient care, which may have changed participants’ practice. In particular, the study simulated a nurse preparing and administering an unfamiliar medicine for the first time. In day-to-day practice, nurses are familiar with most of the medicines they handle, which might reduce both the risk of error and the effect of a user-tested IMG guideline. In addition, to ensure internal validity, nurses were not permitted to ask a colleague for assistance during the simulation and the dose was not double-checked. However, double-checking of intravenous medicines is a commonly adopted practice in UK hospitals to reduce the risk of medication errors, in spite of insufficient evidence of its effectiveness.^34^ As nurses were directly observed, the Hawthorne effect may also have affected their practice and the unfamiliar format of the user-tested guidelines may have caused participants to pay more attention, although evidence suggests that observation does not affect the validity of observational methods for identifying medication administration errors.^22^ These factors (especially the unfamiliarity of the medicine) may explain the high frequency of errors observed in this study (98% of simulations included at least one error of any type) compared with systematic reviews of direct observation studies that have reported error frequencies of 35%–48% in subanalyses of intravenous doses (excluding dose timing errors).^3 4^ Therefore, the absolute error frequencies observed in this study may not be generalisable to day-to-day practice. However, the relative difference in error frequencies between the two groups can be considered generalisable (as both groups were treated equally), particularly in relation to the administration of unusual or complex medicines in NHS hospitals. Finally, blinding was often inadvertently broken by what participants said and did. However, blinding was restored during data analysis.

These results suggest that the user-tested format of the IMG should be adopted and that IMG guidelines should be user-tested. However, the results also have wider implications, suggesting all health systems should consider adopting user-testing for medicines guidelines, particularly those that address high-risk and complex decisions. This will ensure that in addition to being accurate, such guidelines are also usable for their target readership, resulting in a potential improvement in patient safety.^12^

Future research should aim to measure the effects of user-tested guidelines on actual patient care, for example, by focusing on the implementation of the user-tested format of the IMG. In addition, the effects of user-testing have only been examined for two types of medicines guidelines (SPCs and the IMG), and further investigation of its effects on other types of guideline in various healthcare settings is required.

## Conclusion

A user-tested injectable medicines guideline reduced the number of errors and the time taken during the preparation and administration of intravenous medicines. It also resulted in greater confidence and preference among nurses. User-testing has the potential to be widely applied to other types of medicines guidelines, with anticipated improvements in patient safety.
