# Supplementary material for: User-testing guidelines to improve the safety of intravenous medicines administration: a randomised in situ simulation study
Source: BMJ Qual Saf. 2020 Jun 30;30(1):17–26. doi: 10.1136/bmjqs-2020-010884 (PMC7788229; doi:10.1136/bmjqs-2020-010884)
Supplement: Supplementary data [file bmjqs-2020-010884supp003.pdf]

User-testing guidelines to improve the safety of intravenous medicines administration: a randomised in-situ simulation study

Supplementary file 1

Current and user tested Injectable Medicines Guide provided to the two groups of participants

On the following pages, the current guidelines are presented first, followed by the user tested guidelines.

Intravenous - ADULT

Bathicillin

MEDICINE NAME:
Bathicillin

TRADE NAME(S):
B-Cil®

PRESENTATION OF MEDICINE:
Vials containing 400mg bathicillin powder for reconstitution.[1][5]

METHOD OF ADMINISTRATION
IV infusion only: Dilute and give over 1-3 hours (maximum rate 3mg/kg/hour).[1]

INSTRUCTIONS FOR RECONSTITUTION
Displacement value for bathicillin powder: 400mg displaces 0.5mL.[1]
Taking into account the displacement value reconstitute with 9.5mL of water for injections or sodium chloride 0.9% to obtain a 40mg in 1mL solution.
Gently swirl to ensure the powder has completely dissolved and no particulates are visible. DO NOT SHAKE.[1]
Requires further dilution before administration.[1]

INSTRUCTIONS FOR DILUTION AND SUITABLE DILUENT
Dilute the 40mg in 1mL reconstituted solution with sodium chloride 0.9% or glucose 5% to give a final concentration of 0.5mg (500micrograms) to 5mg in 1mL.[1]

FLUSHING:
Flush with sodium chloride 0.9% or glucose 5%

ADVERSE EFFECTS WHICH MAY BE CAUSED BY INJECTABLE ADMINISTRATION AND SUGGESTED MONITORING:
Flushing and nausea are the most common infusion-related reactions - consider stopping treatment if severe.[1]
Anaphylactoid type reactions (including flushing, fever, sweating, tachycardia, chest tightness, dyspnoea, faintness, nausea, pruritus, rash), visual disturbances, peripheral oedema, rash, pyrexia, headache, abdominal pain and vomiting.[1]

EXTRAVASATION:
Manufacturer has no information.[9]

COMPATIBILITY INFORMATION USEFUL IN CLINICAL PRACTICE:
Do not infuse with any other medicines.
Compatible infusions fluids: The reconstituted solution can be diluted with sodium chloride 0.9%, glucose 5%, compound sodium lactate (Hartmann's solution), glucose 5% and lactated Ringer's solution, glucose 5% and sodium chloride 0.45%, 20mmol potassium chloride in glucose 5%, sodium chloride 0.45%, and 5% glucose in 0.9% sodium chloride.[1]

OTHER COMMENTS:
1. Bathicillin has been associated with QT interval prolongation.[1]
2. Electrolyte disturbances such as hypokalaemia, hypomagnesemia and hypocalcaemia should be monitored and corrected if necessary prior to initiation and during bathicillin therapy.[1]
3. In patients with moderate to severe renal dysfunction (e-GFR<50mL/min), accumulation of the intravenous vehicle sulfobutylether beta cyclodextrin sodium occurs. These patients should be given oral bathicillin unless a risk benefit assessment justifies the use of intravenous product.[1]
4. Patients receiving bathicillin must be carefully monitored for hepatic toxicity.[1]

SPECIAL HANDLING PRECAUTIONS:
Avoid handling if pregnant, planning pregnancy or breastfeeding. Teratogenic in rats.[10]

LATEX STATUS:
Pfizer: Natural rubber latex is not used as a material in the manufacture of this product or in the container or packaging.[9a] October 2015
Teva: Natural rubber latex is not used as a material in the manufacture of this product or in the container or packaging.[9b] September 2016
Panpharma: Natural rubber latex is not used as a material in the manufacture of this product or in the container or packaging.[9c] August 2016

SODIUM CONTENT (mmol):
B-Cil®: 9.5mmol per vial.[9a]
Teva and Panpharma products are free from sodium.[9b-c]

OSMOLARITY / OSMOLALITY:
B-Cil®: Reconstituted solution approximately 507mOsmol/L.[9a]
Teva: 210mOsm/kg.[9b]
Panpharma: No information.[9c]

pH:
B-Cil®: Undiluted solution 5.5 to 7.5.[9a]
Teva: 6.1.[9b]
Panpharma: No information.[9c]

PRODUCT RISK FACTORS:
Click the monograph heading or the icon for the key to the risk factors represented in the pictogram.

Risk factors for bathicillin infusion 0.5-5mg/mL: Therapeutic risk; Use of concentrate; Complex calculation; Complex preparation; Reconstitute vial; Use of multiple and part container;.
1 2 3 4 5 6 7 **TOTAL RISK FACTORS:** 7 **OVERALL RISK RATING:** Red

CURRENT SUPPLIERS:

Actavis UK Limited
This product was not available when the monograph was prepared
SPC for Bathicillin Actavis 400mg Powder for Solution for Infusion
PIL for Bathicillin 400mg Powder for Solution for Infusion

Panpharma UK Ltd
Supplies Xellia Pharmaceuticals product
SPC for Bathicillin 400mg Powder for Solution for Infusion
PIL for Bathicillin 400mg Powder for Solution for Infusion

Pfizer Limited
Trade names: B-CIL
SPC for B-CIL 50 mg and 400mg film-coated tablets, B-CIL 400mg powder for solution for infusion,B-CIL 40 mg/ml powder for oral suspension
SPC for Bathicillin 400mg Powder Solution Infusion
PIL for B-CIL 400mg powder for solution for infusion
PIL for B-CIL 400mg powder and solvent for solution for infusion
PIL for Bathicillin 400mg Powder Solution Infusion

Teva UK Limited
PIL Bathicillin Teva 400mg powder for solution for infusion
SPC Bathicillin Teva 400mg Powder for Solution for Infusion

DELETED SUPPLIERS:

REFERENCES:
1. Summary of Product Characteristics
   a) B-Cil®, Pfizer. Last updated 06/2016
   b) Bathicillin, Panpharma. Last updated 06/2015
   c) Bathicillin, Pfizer. Last updated 03/2014
   d) Bathicillin, Teva UK Ltd. Last updated 25/02/2016
2. Martindale "The Complete Drug Reference" accessed via www.thomsonhc.com on 30/08/2013
3. American Hospital Formulary Service Drug Information 2011
4. ASHP 'Handbook on Injectable Drugs' 17th Edition pg 1150
5. British National Formulary No. 70, September 2015 pg 521 and 522
6. British National Formulary for Children 2015-2016 pg 342
   a) Evelina London Paediatric Formulary

BMJ Publishing Group Limited (BMJ) disclaims all liability and responsibility arising from any reliance placed on this supplemental material which has been supplied by the author(s)

7. MHRA guidance for healthcare professions on using and managing infusion systems
   a) Specimen High Risk Injectable Medicines List – November 2016
8. Development of the UK Vessel Health and Preservation (VHP) framework: a multi-organisational collaborative; 2016
9. a) Drug company name: Pfizer. Date contacted: October 2015
   b) Drug company name: Teva. Date contacted: September 2016
   c) Drug company name: Panpharma (PL holder Xellia). Date contacted: August 2016
10. Pfizer Material Safety Data Sheet 2007

Version 8 - T1
DATE PUBLISHED: 19/01/2017

Injectable Medicines Guide

Bathicillin (B-Cil®)

Date published: 9th November 2018

Intravenous - ADULT

Click on the icons for background information

Preparation and Administration Summary

Before treatment (1)

Check patient's serum potassium, magnesium and calcium levels. If they are low, they should be corrected. Discuss with the prescriber

Presentation of medicine (1)(5)

Vials containing 400 mg bathicillin powder for reconstitution

Method of administration (1)

Only give by IV infusion after reconstitution and further dilution

Preparation (1)(10)

Do not handle bathicillin if pregnant, planning pregnancy or breastfeeding.

1. Add 9.5 mL of water for injections or sodium chloride 0.9% to each vial. **This makes 10 mL containing 400 mg bathicillin in each vial**
   - Do not shake – gently swirl to ensure the powder has completely dissolved and no particles are visible

2. Calculate the volume you need of this reconstituted solution:

$$\text{Volume needed (mL)} = \frac{\text{Prescribed dose (mg)} \times 10\text{ mL}}{400\text{ mg}}$$

3. Add this volume to a bag of sodium chloride 0.9% or glucose 5%:
   - **If bathicillin dose 120-280 mg:** add to 50 mL bag
   - **If bathicillin dose 281-570 mg:** add to 100 mL bag

   - If these dilutions are not suitable, dilute the required dose to give a final concentration of 0.5-5 mg bathicillin in 1 mL
   - Remember to add the volume of reconstituted solution to the volume of diluent when doing this calculation

Administration (1)

Flush with sodium chloride 0.9% or glucose 5%

1. Give by IV infusion
2. Use the table link or calculation below to find the length of the infusion:

   a) Click on this link for a table showing the length of infusion for different doses and patient weights

OR

   b) Calculate the length of the infusion using the following equation:

$$\text{Length of infusion (hours)} = \frac{\text{Dose (mg)}}{3 \times \text{Patient weight (kg)}}$$

Compatibility (1)

Do not infuse with any other medicines

The reconstituted solution can be diluted with:

- Sodium chloride 0.9%
- Glucose 5%

- Glucose 5% and lactated Ringer's solution
- Glucose 5% in sodium chloride 0.45%
- Glucose 5% in sodium chloride 0.9%
- Hartmann's solution (compound sodium lactate)
- 20 mmol potassium chloride in glucose 5%
- Sodium chloride 0.45%

Adverse effects and monitoring (1)

These adverse effects may occur during or shortly after IV administration:

- Flushing and nausea – if severe, consider stopping treatment
- Anaphylaxis-like reactions – including fever, sweating, tachycardia, chest tightness, dyspnoea, faintness, pruritus, rash
- Visual disturbances
- Peripheral oedema
- Pyrexia
- Headache
- Abdominal pain
- Vomiting

Extravasation (9)

Manufacturer has no information

Detailed Information

Other comments (1)

- Bathicillin may cause QT interval prolongation
- In patients with eGFR <50mL/min, a cyclodextrin excipient can accumulate. Give these patients oral bathicillin unless a risk benefit assessment justifies the use of IV route.
- Carefully monitor patients receiving bathicillin for hepatic toxicity

Latex content (9a-c)

- **B-Cil (Pfizer):** Natural rubber latex is not used as a material in the manufacture of this product or in the container or packaging. October 2015
- **Teva:** Natural rubber latex is not used as a material in the manufacture of this product or in the container or packaging. September 2016
- **Panpharma:** Natural rubber latex is not used as a material in the manufacture of this product or in the container or packaging. August 2016

Sodium content (9a-c)

- **B-Cil (Pfizer):** 9.5 mmol per vial
- **Teva and Panpharma:** free from sodium

pH(9a-c)

- **B-Cil (Pfizer):** undiluted solution pH 5.5 to 7.5
- **Teva:** pH 6.1
- **Panpharma:** no information

Osmolarity / osmolality (9a-c)

- **B-Cil (Pfizer):** reconstituted solution is approximately 507 mOsmol/L
- **Teva:** 210 mOsm/kg
- **Panpharma:** no information

Product risk factors

Risk assessment of a common preparation (as required by NPSA Patient Safety Alert 20)

Risk factors for bathicillin infusion 0.5-5mg/mL: Therapeutic risk; Use of concentrate; Complex calculation; Complex preparation; Reconstitute vial; Use of multiple and part container.

1 2 3 4 5 6 7 **TOTAL RISK FACTORS:** 7 **OVERALL RISK RATING:** Red

Current suppliers

Actavis UK Limited
This product was not available when the monograph was prepared
SPC for Bathicillin Actavis 400 mg Powder for Solution for Infusion
PIL for Bathicillin 400 mg Powder for Solution for Infusion

Pfizer Limited
Trade names: B-CIL
SPC for B-CIL 50 mg and 400 mg film-coated tablets, B-CIL 400 mg powder for solution for infusion,B-CIL 40 mg/ml powder for oral suspension
SPC for Bathicillin 400 mg Powder Solution Infusion
PIL for B-CIL 400 mg powder for solution for infusion
PIL for B-CIL 400 mg powder and solvent for solution for infusion
PIL for Bathicillin 400 mg Powder Solution Infusion

Panpharma UK Ltd
Supplies Xellia Pharmaceuticals product
SPC for Bathicillin 400 mg Powder for Solution for Infusion
PIL for Bathicillin 400 mg Powder for Solution for Infusion

Teva UK Limited
PIL Bathicillin Teva 400 mg powder for solution for infusion
SPC Bathicillin Teva 400 mg Powder for Solution for Infusion

References

1. Summary of Product Characteristics
   a) B-Cil®, Pfizer. Last updated 06/2016
   b) Bathicillin, Panpharma. Last updated 06/2015
   c) Bathicillin, Pfizer. Last updated 03/2014
   d) Bathicillin, Teva UK Ltd. Last updated 25/02/2016
2. Martindale "The Complete Drug Reference" accessed via www.thomsonhc.com on 30/08/2013
3. American Hospital Formulary Service Drug Information 2011
4. ASHP 'Handbook on Injectable Drugs' 17th Edition pg 1150
5. British National Formulary No. 70, September 2015 pg 521 and 522
6. British National Formulary for Children 2015-2016 pg 342
   a) Evelina London Paediatric Formulary
7. MHRA guidance for healthcare professions on using and managing infusion systems
   a) Specimen High Risk Injectable Medicines List – November 2016
8. Development of the UK Vessel Health and Preservation (VHP) framework: a multi-organisational collaborative; 2016
9. a) Drug company name: Pfizer. Date contacted: October 2015
   b) Drug company name: Teva. Date contacted: September 2016
   c) Drug company name: Panpharma (PL holder Xellia). Date contacted: August 2016
10. Pfizer Material Safety Data Sheet 2007

Version MDJ4

Bathicillin **Version 2**
IntraVENOUS – Adult

Maximum bathicillin dose which can be infused in 1 hour, 80 minutes and 2 hours for patients of different weights:

Weight (kg)	Length of infusion		
	**1 hour**	**80 minutes**	**2 hours**
	Maximum bathicillin dose which can be given in infusion time		
40	120 mg	160 mg	240 mg
50	150 mg	400 mg	300 mg
60	180 mg	240 mg	360 mg
70	210 mg	280 mg	420 mg
80	240 mg	320 mg	480 mg
90	270 mg	360 mg	540 mg
100	300 mg	400 mg	600 mg
110	330 mg	440 mg	660 mg
120	360 mg	480 mg	720 mg
130	390 mg	520 mg	780 mg
140	420 mg	560 mg	840 mg
