# Supplementary material for: User-testing guidelines to improve the safety of intravenous medicines administration: a randomised in situ simulation study
Source: BMJ Qual Saf. 2020 Jun 30;30(1):17–26. doi: 10.1136/bmjqs-2020-010884 (PMC7788229; doi:10.1136/bmjqs-2020-010884)

User-testing guidelines to improve the safety of intravenous medicines administration: a randomised in-situ simulation study

Supplementary file 2

Data collection tools

Structured observation forms

As there were differences between the requirements of the intravenous medicines administration policies of each NHS trust, there were small differences between the structured observation forms used at each research site. The four versions of this form are shown on the following pages.

Trust 1 Observation recording form

Effects of User Testing Injectable Medicines Guidance – Phase 2

Department of Pharmacy & Pharmacology — UNIVERSITY OF BATH

Participant ID: Participant randomisation code: Date: Time taken:

Expected preparation/administration step	No error	Error	Error description/other notes	Error code
Wash hands with soap & water, or alcohol gel				N3
Clean plastic tray with sporicidal detergent wipe, or soap and water. Allow to dry				N3
Wash hands with soap & water or alcohol gel. Put on gloves & apron				N3
Remove seal from bathicillin vial & wipe septum with alcohol wipe. Allow to dry for 30 seconds				N1<br>N3
Draw up reconstituting fluid:				N2
• **WFI or saline 0.9%**				I1
• Fluid has not expired				N4
• Volume = **9.5 mL (8.6-10.5 mL)**				I2
Inject all the reconstituting fluid into the bathicillin vial:				I1<br>I2<br>N2
• Use **bathicillin 400mg vial**				I3<br>N1
• Bathicillin has not expired				N4
With syringe in place, gently swirl until fully dissolved				I6<br>N2
Draw up **9mL (8.6-9.5 mL)** of reconstituted bathicillin solution				I3<br>N1<br>N2
Wipe rubber septum of additive port on infusion bag with alcohol wipe & allow to dry for 30 seconds				N3
Inject **8.6-9.5 mL** reconstituted bathicillin solution into infusion bag through centre of injection port				I3<br>N1<br>N2
• Infusion bag is **saline 0.9% or glucose 5%**				I4

Expected preparation/administration step	No error	Error	Error description/other notes	Error code
• Infusion bag volume is **100/250/500 mL**				I5
• Infusion bag has not expired				N4
Withdraw needle and invert bag at least 5 times to mix				N2
Final infusion for is free of particles, cloudiness and discolouration				N2
Draw up **saline 0.9% or glucose 5% flush**				I8
• Fluid has not expired				N4
Hang infusion bag. Attach administration set to infusion bag and prime. Replace cap on end of administration set. (Close roller ball if using gravity set).				N2
Remove gloves, clean hands with alcohol gel, put on new gloves				N3
Clean hub of cannula with alcoholic chlorhexidine 2% wipe				N3
Flush cannula with **saline 0.9% or glucose 5%** flush				I7<br>I8
Attach giving set to cannula. Open roller clamp/start pump & set infusion rate to give infusion over 2-3 hours (±10%):<br>• **59 mL = 0.30-0.55 mL/min = 6-10 drops/min = 17.9-32.8 mL/hour**<br>• **109 mL = 0.55-1.01 mL/min = 11-20 drops/min = 33.0-60.6 mL/hour**			Rate of infusion:	I7<br>I9<br>N2
Use ANTT throughout, e.g:<br>• Don't touch syringe tips<br>• Don't touch needle<br>• Don't put down syringe with unsheathed needle				N3

Trust 2 Observation recording form

Effects of User Testing Injectable Medicines Guidance – Phase 2

Department of Pharmacy & Pharmacology | UNIVERSITY OF BATH

Participant ID: Participant randomisation code: Date: Time taken:

**Expected preparation/administration step**	**No error**	**Error**	**Error description/other notes**	**Error code**
Clean plastic tray with detergent wipe				N3
Clean hands and put on gloves.				N3
Use ANTT throughout, e.g:<br>• Don't touch syringe tips or needle<br>• Don't put down syringe with unsheathed needle				N3
Remove seal from bathicillin vial & wipe septum with 2% chlorhexidine/70% alcohol wipe. Allow to dry for 30 secs				N1<br>N3
Draw up reconstituting fluid:				N2
• **WFI or saline 0.9%**				I1
• Fluid has not expired				N4
• Volume = **9.5 mL (8.6-10.5 mL)**				I2
Change needle on syringe				N2
Inject all the reconstituting fluid into the bathicillin vial:				I1<br>I2<br>N2
• Use **bathicillin 400mg vial**				I3<br>N1
• Bathicillin has not expired				N4
With syringe in place, gently swirl until fully dissolved				I6<br>N2
Draw up **9mL (8.6-9.5 mL)** of reconstituted bathicillin solution				I3<br>N1<br>N2
Change needle on syringe				N2
Wipe rubber septum of infusion bag additive port with 2% chlorhexidine/70% alcohol wipe & allow to dry for 30 secs				N3

Expected preparation/administration step	No error	Error	Error description/other notes	Error code
Inject **8.6-9.5 mL** reconstituted bathicillin solution into infusion bag through centre of injection port				I3<br>N1<br>N2
• Infusion bag is **saline 0.9% or glucose 5%**				I4
• Infusion bag volume is **100/250/500 mL**				I5
• Infusion bag has not expired				N4
Withdraw needle and invert bag at least 5 times to mix				N2
Final infusion for is free of particles, cloudiness and discolouration				N2
Draw up **saline 0.9% or glucose 5% flush**				I8
• Fluid has not expired				N4
Attach administration set to infusion bag and prime.				N2
Remove gloves, wash hands				N3
Wipe cannula with 2% chlorhexidine/70% alcohol wipe				N3
Put on new gloves				N3
Flush cannula with **saline 0.9% or glucose 5%** flush				I7<br>I8
Attach giving set to cannula. Open roller clamp/start pump & set infusion rate to give infusion over 2-3 hours (±10%):<br>• **59 mL = 0.30-0.55 mL/min = 6-10 drops/min = 17.9-32.8 mL/hour**<br>• **109 mL = 0.55-1.01 mL/min = 11-20 drops/min = 33.0-60.6 mL/hour**			Rate of infusion:	I7<br>I9<br>N2

Trust 3 Observation recording form

Effects of User Testing Injectable Medicines Guidance – Phase 2

Department of Pharmacy & Pharmacology | UNIVERSITY OF BATH

Participant ID: Participant randomisation code: Date: Time taken:

Expected preparation/administration step	No error	Error	Error description/other notes	Error code
Wash hands with soap & water. Put on plastic apron.				N3
Work in a clean aseptic field, e.g. a plastic tray cleaned with a detergent wipe, or soap and water, and allowed to dry				N3
Wash hands with soap & water or alcohol gel. Put on gloves				N3
Remove seal from bathicillin vial & wipe septum with alcohol wipe. Allow to dry.				N1<br>N3
Draw up reconstituting fluid:				N2
• **WFI or saline 0.9%**				I1
• Fluid has not expired				N4
• Volume = **9.5 mL (8.6-10.5 mL)**				I2
Inject all the reconstituting fluid into the bathicillin vial:				I1<br>I2<br>N2
• Use **bathicillin 400mg vial**				I3<br>N1
• Bathicillin has not expired				N4
Gently swirl until fully dissolved				I6<br>N2
Draw up **9mL (8.6-9.5 mL)** of reconstituted bathicillin solution				I3<br>N1<br>N2
Inject **8.6-9.5 mL** reconstituted bathicillin solution into infusion bag through centre of injection port				I3<br>N1<br>N2

Expected preparation/administration step	No error	Error	Error description/other notes	Error code
• Infusion bag is **saline 0.9% or glucose 5%**				I4
• Infusion bag volume is **100/250/500 mL**				I5
• Infusion bag has not expired				N4
Draw up **saline 0.9% or glucose 5% flush**				I8
• Fluid has not expired				N4
Clean hub of cannula with alcoholic chlorhexidine 2% wipe				N3
Flush cannula with **saline 0.9% or glucose 5%** flush				I7<br>I8
Administer infusion over 2-3 hours (±10%):<br>• **59 mL = 0.30-0.55 mL/min = 6-10 drops/min = 17.9-32.8 mL/hour**<br>• **109 mL = 0.55-1.01 mL/min = 11-20 drops/min = 33.0-60.6 mL/hour**			Rate of infusion:	I7<br>I9<br>N2
Use ANTT throughout, e.g:<br>• Don’t touch syringe tips<br>• Don’t touch needle<br>• Don’t put down syringe with unsheathed needle				N3

Trust 4 Observation recording form

Effects of User Testing Injectable Medicines Guidance – Phase 2

Department of Pharmacy & Pharmacology | UNIVERSITY OF BATH

Participant ID: Participant randomisation code: Date: Time taken:

Expected preparation/administration step	No error	Error	Error description/other notes	Error code
Wash hands				N3
Clean plastic tray with universal cleaning and disinfecting wipe. Allow to dry.				N3
Wash hands with soap & water or alcohol gel. Put on gloves & apron				N3
Remove seal from bathicillin vial & clean septum				N1<br>N3
Draw up reconstituting fluid:				N2
• **WFI or saline 0.9%**				I1
• Fluid has not expired				N4
• Volume = **9.5 mL (8.6-10.5 mL)**				I2
Inject all the reconstituting fluid into the bathicillin vial:				I1<br>I2<br>N2
• Use **bathicillin 400mg vial**				I3<br>N1
• Bathicillin has not expired				N4
Gently swirl until fully dissolved				I6<br>N2
Draw up **9mL (8.6-9.5 mL)** of reconstituted bathicillin solution				I3<br>N1<br>N2
Inject **8.6-9.5 mL** reconstituted bathicillin solution into infusion bag through centre of injection port				I3<br>N1<br>N2

Expected preparation/administration step	No error	Error	Error description/other notes	Error code
• Infusion bag is **saline 0.9% or glucose 5%**				I4
• Infusion bag volume is **100/250/500 mL**				I5
• Infusion bag has not expired				N4
Draw up **saline 0.9% flush**				I8
• Flush volume is 5 mL				N5
• Fluid has not expired				N4
Remove gloves, clean hands with alcohol gel, put on new gloves				N3
Clean hub of cannula with alcoholic chlorhexidine 2% wipe for 20 seconds and allow to dry completely				N3
Flush cannula with 5ml **saline 0.9%** flush				I7<br>I8
Administer infusion over 2-3 hours (±10%):<br>• **59 mL = 0.30-0.55 mL/min = 6-10 drops/min = 17.9-32.8 mL/hour**<br>• **109 mL = 0.55-1.01 mL/min = 11-20 drops/min = 33.0-60.6 mL/hour**			Rate of infusion:	I7<br>I9<br>N2
Use ANTT throughout, e.g:<br>• Don't touch syringe tips<br>• Don't touch needle<br>Don't put down syringe with unsheathed needle				

Participant characteristics and feedback data collection form

Questions 13-20 on the final page make up the modified Provider Decision Process Assessment Instrument (mPDPAI) questionnaire. Questions 21 and 22 are the quality and satisfaction questions used to validate the mPDPAI. Question 22 is the guidelines preference question.

Department of Pharmacy & Pharmacology | UNIVERSITY OF BATH

Observation data collection form

Effects of User Testing Injectable Medicines Guidance – Phase 2

Completed by researcher:

**Hospital:**	Site 1	Site 2	Site 3	Site 4
**Participant randomisation code:**				

Please answer the following questions (please circle one answer or fill in the box):

**Have you already taken part in this study on another day?**	Yes		No
**What best describes your gender?**	Female	Male	Prefer not to say
	Prefer to self-describe:		
**How many years old are you?**			
**Is English your first language?**	Yes	No	
**Are you currently a qualified nurse or midwife registered with the Nursing and Midwifery Council**	Yes	No	
**For how long have you worked as a qualified nurse or midwife?**			
**Are you currently authorised to prepare and administer IV medicines in this hospital?**	Yes	No	
**For how long have you been authorised to administer IV medicines?**			
**Over the last six months (or since you were authorised), on approximately what percentage of your shifts have you prepared or administered an IV medicine?**	%		
**Have you used the Medusa IV guide before?**	I use it regularly at the moment		
	I used it regularly in the past, but not at the moment		
	I've seen it before, but not used it often		
	I haven't seen it before		

Please give this sheet back to the researcher

11th January, 2019 Version: 1.0 IRAS ID: 242505

Please answer the questions below to tell us about your experience of making and giving bathicillin (please circle one answer):

13	**It was hard to decide how to make and give bathicillin**	Strongly agree	Agree	Neither agree nor disagree	Disagree	Strongly disagree
14	**I was unsure how to make and give bathicillin**	Strongly agree	Agree	Neither agree nor disagree	Disagree	Strongly disagree
15	**It was clear how to make and give bathicillin**	Strongly agree	Agree	Neither agree nor disagree	Disagree	Strongly disagree
16	**When deciding how to make and give bathicillin, I felt I did not know enough about the alternative ways of doing this**	Strongly agree	Agree	Neither agree nor disagree	Disagree	Strongly disagree
17	**I had trouble deciding how to make and give bathicillin because important information was not available**	Strongly agree	Agree	Neither agree nor disagree	Disagree	Strongly disagree
18	**It was easy to identify all the information needed to make and give bathicillin**	Strongly agree	Agree	Neither agree nor disagree	Disagree	Strongly disagree
19	**I am satisfied with how I made and gave bathicillin**	Strongly agree	Agree	Neither agree nor disagree	Disagree	Strongly disagree
20	**I am satisfied that the process used to decide how to make and give bathicillin was as good as it could be**	Strongly agree	Agree	Neither agree nor disagree	Disagree	Strongly disagree

21	**Please rate the quality of how you made and gave the dose of bathicillin**	0	1	2	3	4	5	6	7	8	9	10
		Worst → Best										

22	**How would you feel if you made every IV medicine for the rest of your career how you made bathicillin?**	Terrible	Unhappy	Dissatisfied	Neutral	Satisfied	Pleased	Delighted

Now ask the researcher to show you two IV guides (Guide X and Guide Y). You will have already used one of these guides - please do not tell the researcher which one this was! Please look at both guides and then answer the following question:

23	**Which guide do you prefer?**	I strongly prefer guide X	I prefer guide X	I somewhat prefer guide X	Same preference for guide X or Y	I somewhat prefer guide Y	I prefer guide Y	I strongly prefer guide Y

11th January, 2019 Version: 1.0 IRAS ID: 242505

Images of simulated medicine

Participants selected a vial from a box that contained twenty-five identical placebo vials (Dummy-Ject Powder C, MockMeds, Houston, Texas). Twenty-three of these vials were labelled as the unexpired medicine required for the prescribed dose:

For simulation research only
Bathicillin for injection
400 mg per vial
Expiry: 31 Dec 2019

One of these vials was labelled as an alternative invented medicine name:

For simulation research only
Unimycin for injection
400 mg per vial
Expiry: 31 Dec 2019

The remaining one vial was labelled as the required medicine, but with an expiry date that had passed:

For simulation research only
Bathicillin for injection
400 mg per vial
Expiry: 31 Oct 2017

The labelled vials are shown below:

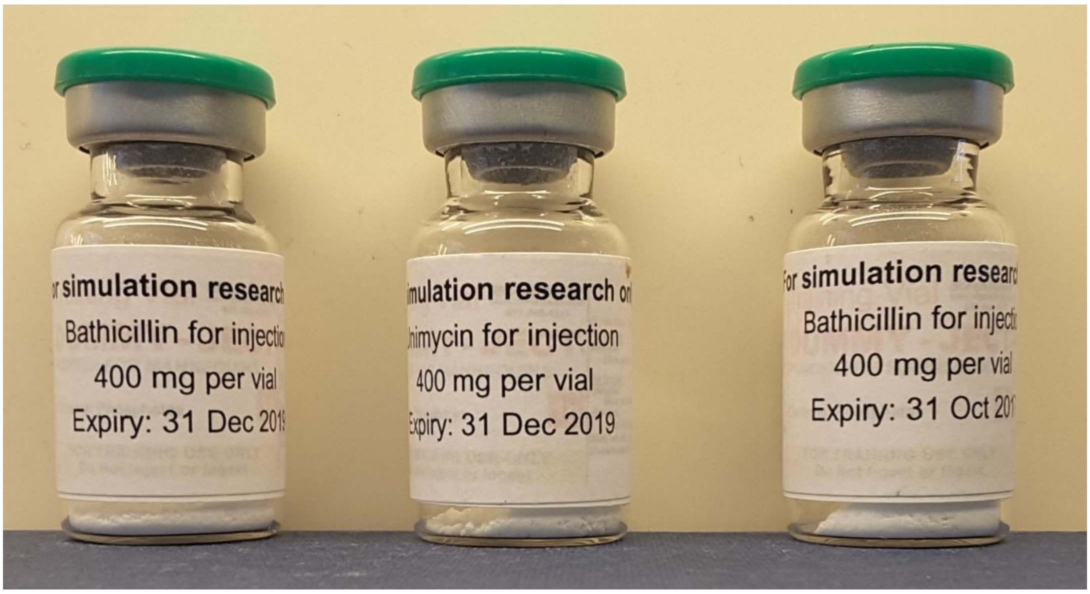

Supplement: Supplementary data [file bmjqs-2020-010884supp001.pdf]
