# Supplementary material for: User-testing guidelines to improve the safety of intravenous medicines administration: a randomised in situ simulation study
Source: BMJ Qual Saf. 2020 Jun 30;30(1):17–26. doi: 10.1136/bmjqs-2020-010884 (PMC7788229; doi:10.1136/bmjqs-2020-010884)
Supplement: Supplementary data [file bmjqs-2020-010884supp002.pdf]

User-testing guidelines to improve the safety of intravenous medicines administration: a randomised in-situ simulation study

Supplementary file 3

Additional Tables, Figures and Data

Table 4: Definitions of error types and categorisation as "IMG-related errors" or "non-IMG-related errors"

Error code	Error type	Definition
IMG-related errors – an error in a process that required use of information from the IMG		
I1	Wrong reconstituting fluid	The dose is reconstituted with a different fluid to that specified in the IMG guidelines
I2	Wrong reconstituting fluid volume	The dose is reconstituted with a volume of fluid which differs from that specified in the IMG guidelines by ≥10%
I3	Dose discrepancy	The administered dose differs from the prescribed dose by ≥5%[a]
I4	Wrong diluent	The dose is infused in a different diluent to that specified in the IMG guidelines
I5	Wrong diluent volume	The dose is infused in a different volume of diluent to that specified in the IMG guidelines
I6	Incorrect technique (IMG-related)	The dose is prepared or administered in a way that does not meet the requirements specified by the IMG guidelines, e.g. the vial is shaken when the guidelines specify "do not shake".
I7	Wrong route	The dose is administered via a different route to that specified on the medication order
I8	Flush error	The intravenous cannula is not flushed in accordance with hospital policy before administration of the dose. (Any requirements for a flush after administration were not relevant, as the infusion was not observed for its entire duration).
I9	Rate discrepancy	The dose is administered at a rate that differs from that specified in the IMG guidelines by ≥10%
I10	Infusion expiry error	An infusion is labelled with an expiry date and time that is not in accordance with the IMG guidelines
I11	Other IMG related error	Any IMG related error which does not fit one of the above categories, including the participant not being sufficiently confident to finish the simulation without assistance from a colleague (included in analysis as delayed administration)
Non-IMG-related errors – an error in a process that did not require use of information from the IMG		
N1	Wrong medication	A different medication to that specified on the medication order is administered
N2	Incorrect technique (non-IMG related)	The dose is prepared or administered in a way that does not meet the requirements of hospital policy, e.g. incorrect mixing technique, air in the syringe, where these details are not specified in the IMG guidelines.
N3	Non-aseptic technique	Breach of the aseptic technique policy of the hospital during the preparation or administration of the dose (e.g. hands not washed, vials/additive ports not swapped with an alcohol wipe)
N4	Expired ingredients	Use of expired medicine, reconstituting fluid, diluent or flush.
N5	Other non-IMG related error	Any non-IMG related error which does not fit the above categories

[a]A figure of 5% was chosen to ensure that an error is recorded when the participant does not account for the displacement value, a common error during the previous user-testing study. A threshold of 5% has been used in a number of previous studies.
IMG = Injectable Medicines Guide

Figure 3: Kaplan-Meier Curve showing the times taken to prepare and administer the dose of voriconazole in each group

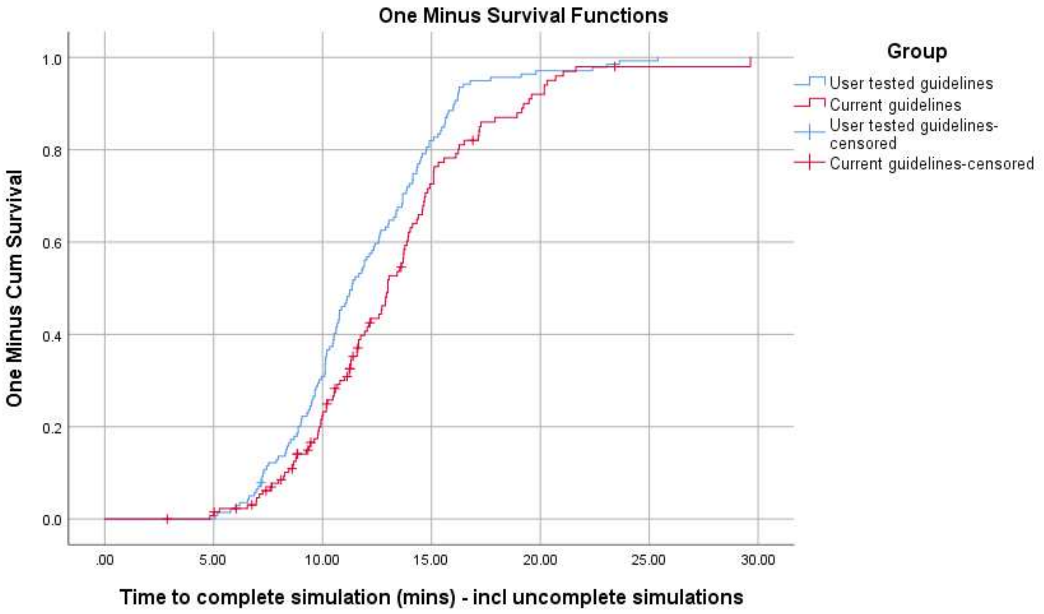

Psychometric Properties of the Modified Provider Decision Process Assessment Instrument (mPDPAI)

Item homogeneity of the mPDPAI was evaluated by calculating Spearman's rank correlation coefficients between each item and a revised mDCS calculated by removing that item from the total score:

Table 5: Psychometric properties of the modified provider decision process assessment instrument (mPDPAI)

Item removed from the mPDPAI	Spearman's rank correlation coefficient
It was hard to decide how to make and give bathicillin	0.85
I was unsure how to make and give bathicillin	0.83
It was clear how to make and give bathicillin	0.83
When deciding how to make and give bathicillin, I felt I did not know enough about the alternative ways of doing this	0.63
I had trouble deciding how to make and give bathicillin because important information was not available	0.79
It was easy to identify all the information needed to make and give bathicillin	0.86
I am satisfied with how I made and gave bathicillin	0.75
I am satisfied that the process used to decide how to make and give bathicillin was as good as it could be	0.80

Inter-item reliability of the mPDPAI was confirmed by a Cronbach's alpha of 0.95.

Construct validity of the mPDPAI was confirmed by the strong negative correlation coefficients between the mDCS and participants' self-rating of quality (-0.78), and the mDCS and participants' rating of their feelings about the process (-0.82). A weak positive correlation coefficient was found between mDCS score and time to complete the simulation (0.23). Mixed effects logistic regression with any IMG-related error as the dependent variable, NHS trust as a random effect, and mDCS and other participants characteristics as fixed-effect covariates found an mDCS odds ratio of

1.12 (95% confidence interval 1.08-1.17), confirming that participants with a higher mDCS were more likely to make an IMG-related error.
